# PD-1-positive Natural Killer Cells have a weaker antitumor function than that of PD-1-negative Natural Killer Cells in Lung Cancer

**DOI:** 10.7150/ijms.47701

**Published:** 2020-07-19

**Authors:** Chao Niu, Min Li, Shan Zhu, Yongchong Chen, Lei Zhou, Dongsheng Xu, Jianting Xu, Zhaozhi Li, Wei Li, Jiuwei Cui

**Affiliations:** 1 Department of Cancer Center, The First Hospital of Jilin University, Changchun 130021, China.; 2 Department of Translational Medicine, The First Hospital of Jilin University, Changchun 130021, China.

**Keywords:** natural killer cells, programmed death-1, cytotoxicity, lung cancer

## Abstract

Antibodies targeting the immune checkpoint inhibitor, programmed cell death 1 (PD-1), have provided a breakthrough in the treatment of lung cancer. However, the function of PD-1 in natural killer (NK) cells of cancer patients remains unclear. Herein, we analyzed the expression of PD-1 on the NK cells in the peripheral blood of patients with lung cancer and found that the level of PD-1^+^ NK cells in patients was significantly higher than that in healthy individuals. Moreover, these PD-1^+^ NK cells demonstrated a weaker ability to secrete interferon-gamma (INF-γ), granzyme B, and perforin, and exhibited lower CD107a expression. Importantly, in patients with lung cancer, the percentage of PD-1^+^ NK cells was significantly positively correlated with the concentration of IL-2 in the plasma, which was also higher than that in healthy individuals. In addition, IL-2 could increase the expression of PD-1 on NK cells* in vitro*, indicating that high IL-2 level in the plasma is largely responsible for the abundance of PD-1^+^ NK cells in patients with lung cancer. These findings demonstrate intriguing mechanisms for understanding the expression of PD-1 on NK cells and the function of PD-1^+^ NK cells in lung cancer. This study confirms and extends previous studies demonstrating that PD-1 can negatively regulate the antitumor function of NK cells.

## Introduction

Cancer is a major disease that has become a threat to human health in recent years. Lung cancer causes about 27% of all cancer deaths and has the highest morbidity and mortality among malignant diseases worldwide 1, 2. Immune checkpoint inhibitor, programmed death (PD)-1, is mainly expressed on the membranes of activated T cells and monocytes 3. PD-1 performs an essential function in the tumor immune escape 4-7. The main ligand of PD-1 is program death ligand-1 (PD-L1), which is expressed not only on the membrane of normal cells but also of lung cancer cells 8. By blocking the interactions of PD-1 and PD-L1, the immune system can be reactivated to fight lung cancer 7. PD-1 inhibitors, such as pembrolizumab and nivolumab, have been approved by regulatory authorities for the treatment of non-small cell lung cancer 9, 10. Although therapeutic strategies targeting the interactions of PD-1 and PD-L1 have recently shown promising clinical outcomes in lung cancer, challenges remain. Not all patients will benefit from these antibody drugs, and the mechanism still requires further investigations.

Immune cell therapy is a vital part of tumor therapy. Natural killer (NK) cells are critical innate immune cells 11, which can directly kill tumor cells by secreting perforin and granzyme and through an antibody-dependent cell-mediated cytotoxicity effect 12. They can also inhibit the proliferation of tumor cells by secreting interferon-gamma (IFN-γ) 13. NK cells play an essential role in lung cancer progression; the infiltration of NK cells in tumor tissues is related to a favorable prognosis in patients with lung cancer 14-16. Moreover, the cytotoxicity and IFN-γ secretion abilities are significantly lower in peripheral blood NK cells from patients with lung cancer than in controls 17. Hence, exploring the mechanisms of NK cell dysfunction is very meaningful for the treatment of lung cancer.

PD-1 mediates the functional defects in NK cells found in some cancers, such as Kaposi sarcoma and multiple myeloma 18-20. Blocking signal transmission between PD-1 and PD-L1 has been shown to restore the antitumor function of NK cells 21, 22. However, the characteristics of PD-1^+^ NK cells in lung cancer have not been evaluated. Therefore, whether the dysfunction of NK cells is associated with PD-1 expression in lung cancer requires deep investigation.

Recently, extensive studies on the functions of various cytokines in tumor development have been performed. The concentration of IFN-γ in plasma has been related to the response to PD-1 inhibitors in lung cancer, indicating that cytokines may be pivotal for the development of lung cancer 23. Whether plasma cytokines in lung cancer patients will affect the expression of PD-1 in NK cells, and whether they are related to NK cell dysfunction are worth extensive studies.

Therefore, in this study, we determined the ratio of PD-1^+^ NK cells and the level of cytokines in the plasma of lung cancer patients and ascertained their correlation. We also evaluated the antitumor functions of PD-1^+^ NK cells.

## Materials and methods

### Volunteer enrollment

After our hospital's ethics committee approved the study, sixteen newly diagnosed lung cancer patients and sixteen healthy adult volunteers were enrolled, and informed consent was obtained from all participants. In order to avoid the effect of the age and gender, the age and gender of the healthy adult volunteers are matched with that of lung cancer patients. Additionally, the study strictly complied with the Declaration of Helsinki. The characteristics of all participants are listed in Table 1 and Table 2, respectively.

### Phenotypic analyses of blood cells

Peripheral blood of all participates was extracted using heparin anti-coagulation venipuncture tubes. Blood (200 μL) was incubated with mouse mAbs against human CD56 (FITC), CD3 (PerCP) (BD Biosciences, San Jose, CA, USA), and PD-1 (APC) (BioLegend, San Diego, CA, USA) for 15 min at 23 °C. The control groups were stained with isotype-matched antibodies. Red blood cell lysis buffer was added to the tubes containing red blood cells to lyse them. After incubating at 23 °C for 5 min and one wash with PBS, cells were detected using a BD FACSCalibur cell analyzer (BD Biosciences). Data was then analyzed using FlowJo software (Tree Star Inc., Ashland, OR, USA).

### Isolation of peripheral blood mononuclear cells

After separation from blood cells via centrifugation at 1,800 × *g* and 23 °C for 10 min, the plasma was collected for cytokine detection. Blood cells were suspended in normal saline and centrifuged at 800 × *g* and 23 °C for 30 min to obtain peripheral blood mononuclear cells (PBMCs) using Ficoll (Nycomed Pharma AS, Oslo, Norway).

### Cytotoxicity assay

K562 cells were labelled with Calcein-AM (Dojindo Laboratories, Kumamoto, Japan) at 37 °C for 30 min. After washing with PBS, 5×10^3^ Calcein-AM-labelled K562 cells were added to a 96-well plate with 100 μL RPMI-1640 medium (Gibco, Grand Island, NY, USA). PBMCs were added to the wells at effector-target ratios of 20:1 in 100 μL medium. Spontaneous release was obtained by incubating the target cells in medium alone, and maximum release was obtained after treatment with 1% Triton X-100. All experiments were performed in triplicate wells. Cytotoxicity was calculated according to the following formula: [(experimental release - spontaneous release)/(maximum release - spontaneous release)] × 100%^24^.

### PD-1 detection on cytokine stimulated NK cells

PBMCs were incubated with or without interleukin (IL)-2 for 2 days. The cells were then incubated with mouse mAbs against human CD56 (FITC), CD3 (PerCP), and PD-1 (APC). The control groups were stained with isotype-matched antibodies. After one wash with PBS, the cells were detected by the FACSCalibur cell analyzer. The data were analyzed as above.

### Plasma cytokine analysis

Plasma from all participants was measured in duplicate wells using the Milliplex human cytokine/chemokine 96-well plate assay (Millipore, Billerica, MA, USA). The plates were read on a Luminex 200 analyzer (Luminex Corporation, Austin, TX, USA). Five analytes were measured: IL-2, tumor necrosis factor-alpha (TNF-α), IL-10, IFN-γ, and IL-6.

### Intracellular staining analysis

Intracellular staining was carried out according to the manual of the BD Cytofix/Cytoperm™ kit (BD Biosciences). Briefly, PBMCs were harvested and adjusted to 1 × 10^6^ cells/mL. The cells were incubated with 0.1% GolgiStop (BD Biosciences) in an incubator for 4 h. The cells were incubated with mouse mAbs against human CD56 (FITC), CD3 (PerCP), and PD-1 (APC). These were followed by intracellular staining with mouse mAbs against human perforin (PE), granzyme B (PE), or IFN-γ (PE) (BD Pharmingen, San Jose, CA, USA), as well as the isotype control antibodies. After one wash with PBS, the cells were detected by the FACSCalibur cell analyzer. The data were analyzed as above.

### Degranulation assay

As previously described, the expression of CD107a was used to assess the cytotoxicity ability of NK cells 25, 26. PBMCs and K562 cells were incubated at a ratio of 10:1. A mouse mAb against human CD107a-PE (BD Biosciences) and an isotype control antibody were added to the cells. Following stimulation with K562 cells for 1 h, 0.1% GolgiStop was added. After another 3 h incubation, the cells were collected and stained with mouse mAbs against human CD3 (PerCP), CD56 (FITC), and PD-1 (APC). After one wash with PBS, the cells were detected and analyzed as above.

### Statistical analysis

The proportions of cells were analyzed and compared using the paired *t*-test or repeated measures ANOVA and Tukey's multiple comparison test. Spearman's test was used for correlation analysis. GraphPad Prism 5 software was used for data analysis (GraphPad Software, San Diego, CA).

## Results

### Lung cancer patient NK cells exhibit diminished antitumor function

**Figure 1:**
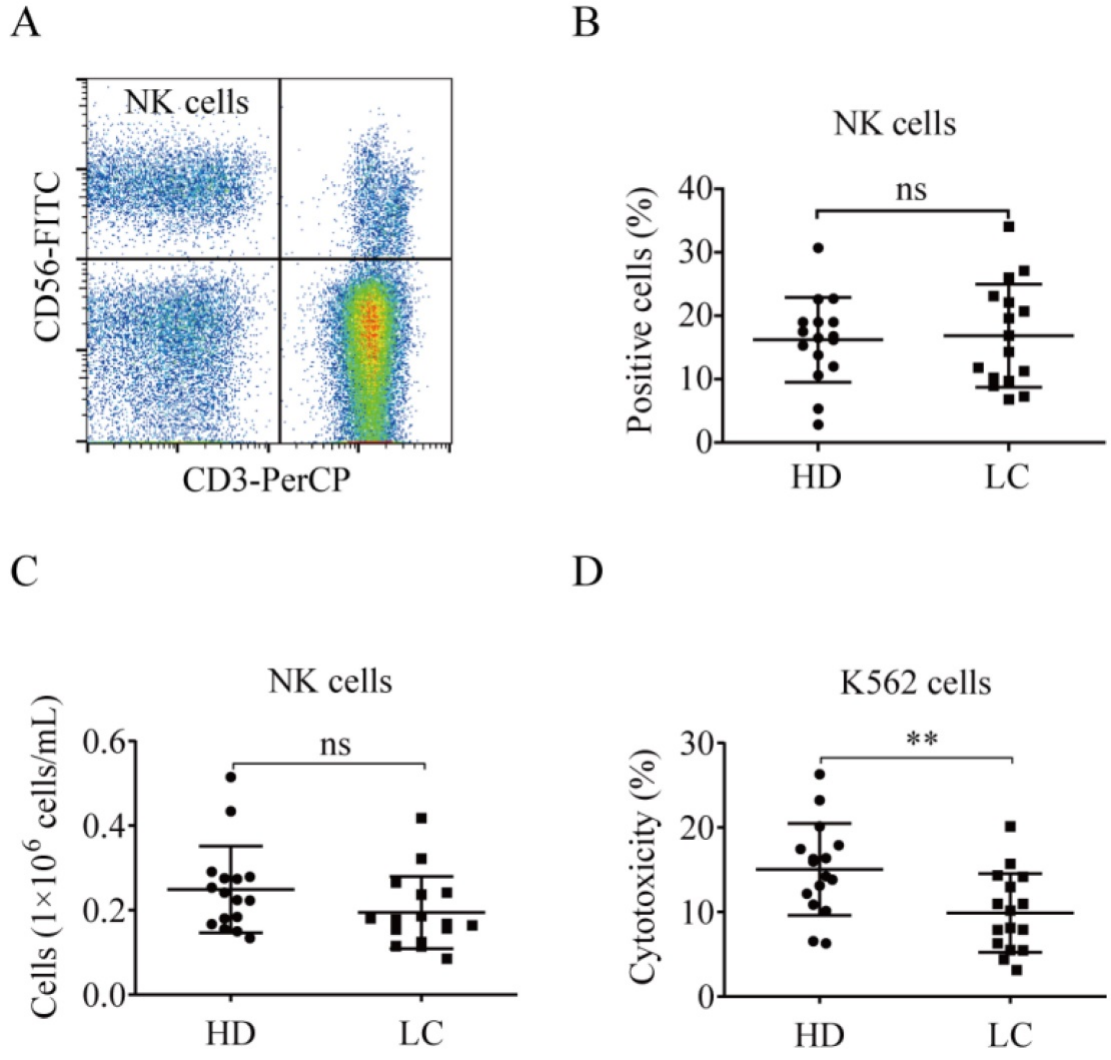
** NK cells in lung cancer patients demonstrate reduced antitumor function.** (**A**) Representative flow cytometry analysis of the expression levels of CD3^-^CD56^+^ NK cells. (**B**) Graph showing the percentages of NK cells in the blood of lung cancer patients (LC) and healthy donors (HD). Data were analyzed and compared using the paired *t*-test; ns: not significant. (**C**) Numbers of NK cells in the peripheral blood of LC and HD. The absolute numbers of NK cells were obtained by multiplying PBMCs per milliliter of peripheral blood by the percentage of NK cells. Data were analyzed and compared using the paired *t*-test. ns: not significant. (**D**) Cytotoxicity of NK cells from LC and HD against K562 cells. Data were analyzed and compared using the paired *t*-test; **p < 0.01.

NK cell proportions were similar between the lung cancer and healthy control groups (Fig. 1A and B). The number of peripheral blood NK cells in lung cancer patients was nearly the same as in the healthy donors [(0.19 ± 0.09)×10^6^ cells/mL vs. (0.25 ± 0.10)×10^6^ cells/mL, *p* = 0.1134; Fig. 1C]. The PBMCs from lung cancer patients and healthy donors were tested for NK cell cytotoxicity. The antitumor function of the lung cancer NK cells was significantly lower than that of the healthy donors (9.88% ± 4.66% *vs.* 15.04% ± 5.42%, *p* = 0.0071; Fig. 1D).

### Lung cancer patients have high PD-1^+^ NK cell levels

**Figure 2:**
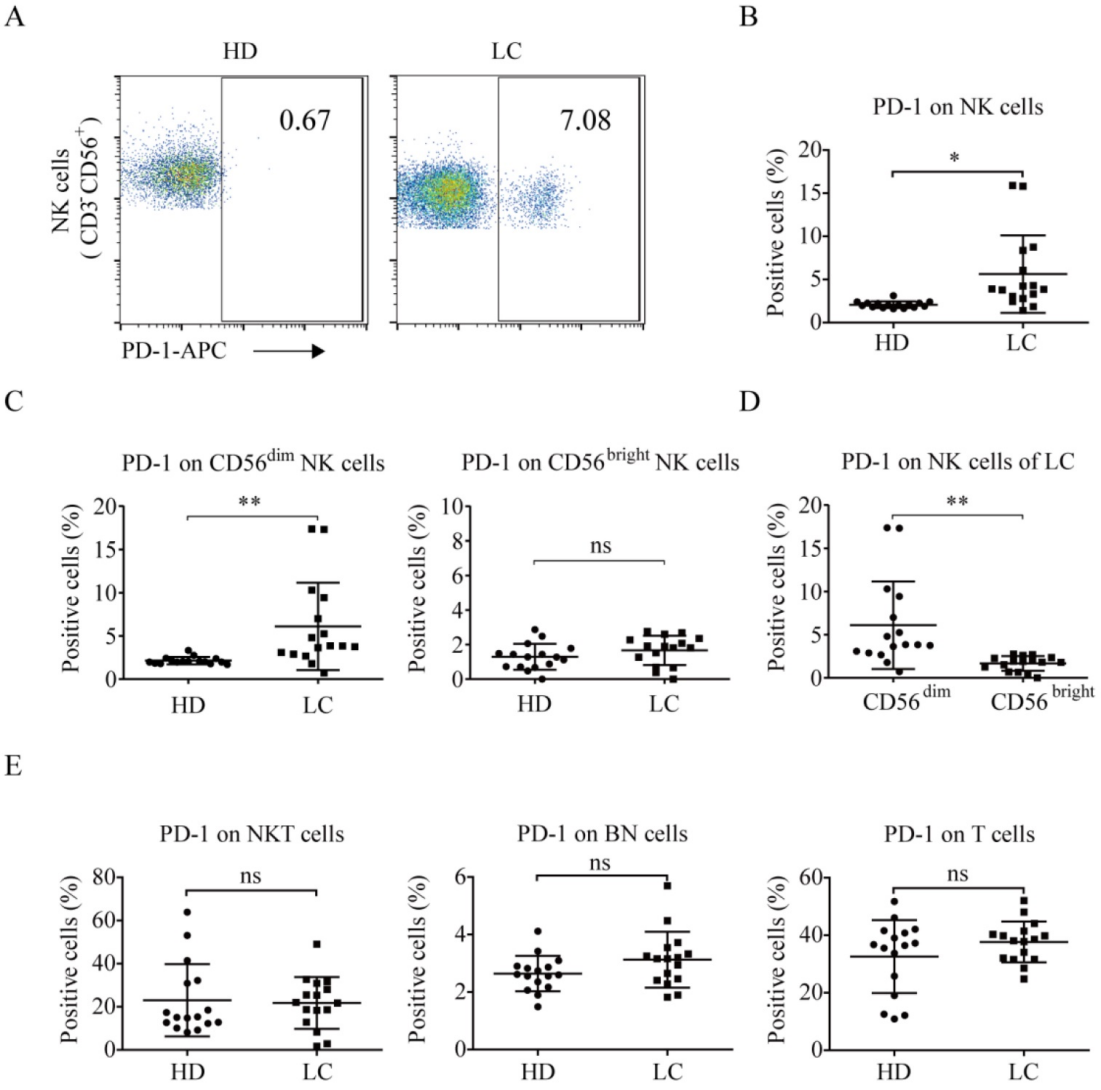
** PD-1 expression in NK cells of lung cancer patients and healthy individuals.** Peripheral blood of healthy donors and lung cancer patients was incubated with mouse mAbs against human CD56, CD3, and PD-1. After the lysis of red blood cells, the cells were analyzed by a flow cytometer. (**A**) Representative flow cytometry analysis of PD-1 expression on NK (CD3^-^CD56^+^) cells in lung cancer patients (LC) and healthy donors (HD). (**B**) Graph showing the percentage of PD-1 expression on the NK cells of 16 HD and 16 LC. Data were analyzed and compared using the paired *t*-test; *p < 0.05. (**C, D**) Graph showing the percentage of PD-1 expression on the CD56^ dim^ NK cells and CD56^bright^ NK cells of 16 HD and 16 LC. Data were analyzed and compared using the paired *t*-test; **p < 0.01, ns: not significant. (**E**) Graph showing the percentage of PD-1 expression on the CD3^+^CD56^+^ NKT cells, CD3^-^CD56^-^ (both negative, BN) cells, and T cells of 16 HD and 16 LC. Data were analyzed and compared using the paired *t*-test. ns: not significant.

PD-1 is rarely expressed on the NK cells of healthy individuals 19, 20, 22, 27. We found that the lung cancer group exhibited higher PD-1^+^ NK cell levels than the healthy donors (5.62% ± 4.49% *vs.* 2.08% ± 0.38%, *p* = 0.0037; Fig. 2A and 2B). Additionally, CD56^dim^ NK cells in the peripheral blood of the lung cancer group exhibited a significantly higher level of PD-1 expression than that of the healthy donors (6.11% ± 5.07% *vs.* 2.14% ± 0.42%, *p* = 0.0055; Fig. 2C). However, there was no difference in the percentage of PD-1^+^ CD56^bright^ NK cells between lung cancer group and healthy donors (1.67% ± 0.85% *vs.* 1.30% ± 0.75%, *p* = 0.2655). In the lung cancer group, the PD-1^+^ NK cells were mainly CD56^dim^ NK cells, not CD56^bright^ NK cells (6.11% ± 5.07% *vs.* 1.67% ± 0.85%, *p* = 0.0024; Fig. 2D). Moreover, the patients and healthy donors had the same ratios of PD-1 expression on CD3^+^CD56^+^ NKT cells (21.79% ± 12.01% *vs.* 23.01% ± 16.74%, *p* = 0.8155), CD3^-^CD56^-^ (both negative, BN) cells (3.13% ± 0.97% *vs.* 2.64% ± 0.62%, *p* = 0.1044), and CD3^+^CD56^-^ T cells (37.64% ± 7.09% *vs.* 32.57% ± 12.69%, *p* = 0.1727; Fig. 2E).

### PD-1^+^ NK cells exhibited diminished antitumor function and IFN-γ secretion compared to PD-1^-^ NK cells

**Figure 3:**
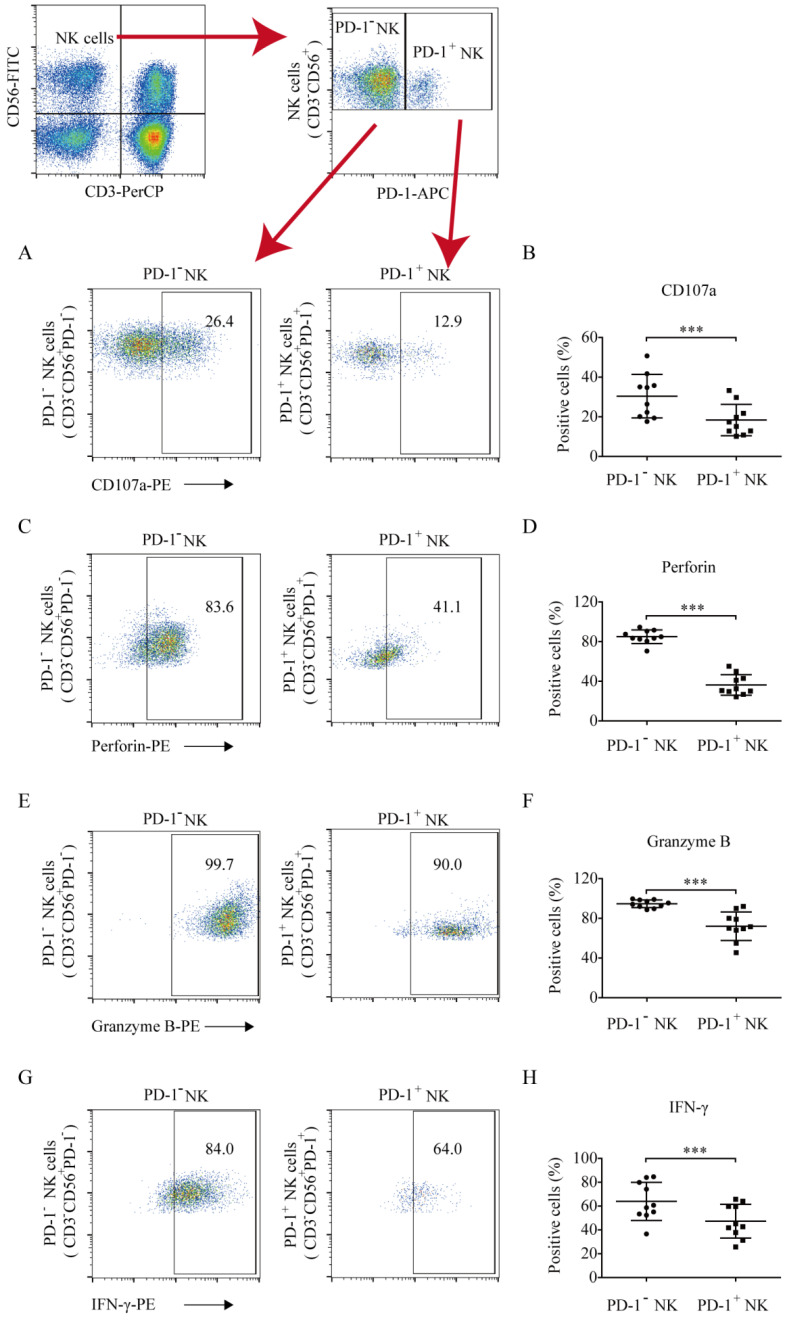
** The levels of CD107a, perforin, granzyme B, and IFN-γ production in PD-1^+^ and PD-1^-^ NK cells.** The cells were collected, washed, and stained with CD56-FITC, CD3-PerCP, and PD-1-APC (to identify PD-1^+^ and PD-1^-^ NK cells) and with PE-labelled CD107a, perforin, granzyme B, or IFN-γ (to identify their expression in these cell populations). (**A, C, E, G**) Flow cytometry analysis of the PD-1^+^ and PD-1^-^ NK cells of one representative patient that were stained with (A) CD107a-PE, (C) perforin-PE, (E) granzyme B-PE, (G) or IFN-γ-PE. (**B, D, F, H**) Comparison of the expression of CD107a, perforin, granzyme B, and IFN-γ in PD-1^+^ and PD-1^-^ NK cells in ten lung cancer patients who had a higher proportion of PD-1^+^ NK cells. Data were analyzed and compared using the paired *t*-test; ***p < 0.001.

To quantify the antitumor function of PD-1 positive and negative NK cells against hematologic tumors, the CD107a degranulation and perforin and granzyme B levels were analyzed. Compared to PD-1^-^ NK cells, PD-1^+^ NK cells exhibited low levels of CD107a (18.38% ±7.91% *vs.* 30.39% ± 10.97%, *p* = 0.0004; Fig. 3A and 3B), perforin (36.37% ±10.39% *vs.* 85.02% ± 6.82%, *p* < 0.0001; Fig. 3C and 3D), and granzyme B (72.08% ± 14.39% *vs.* 94.70% ± 3.89%, *p* = 0.0002; Fig. 3E and 3F) secretion, indicating that PD-1^+^ NK cells exhibited weaker antitumor activity. The IFN-γ percentages in PD-1 positive and negative NK cells were 47.35% ± 14.12% and 63.96% ± 15.98%, respectively. PD-1^+^ NK cells demonstrated a weaker ability to secrete IFN-γ than that of PD-1**^-^** NK cells (Fig. 3G and 3H).

### PD-1^+^ NK cell level and IL-2 plasma concentration were positively related

**Figure 4:**
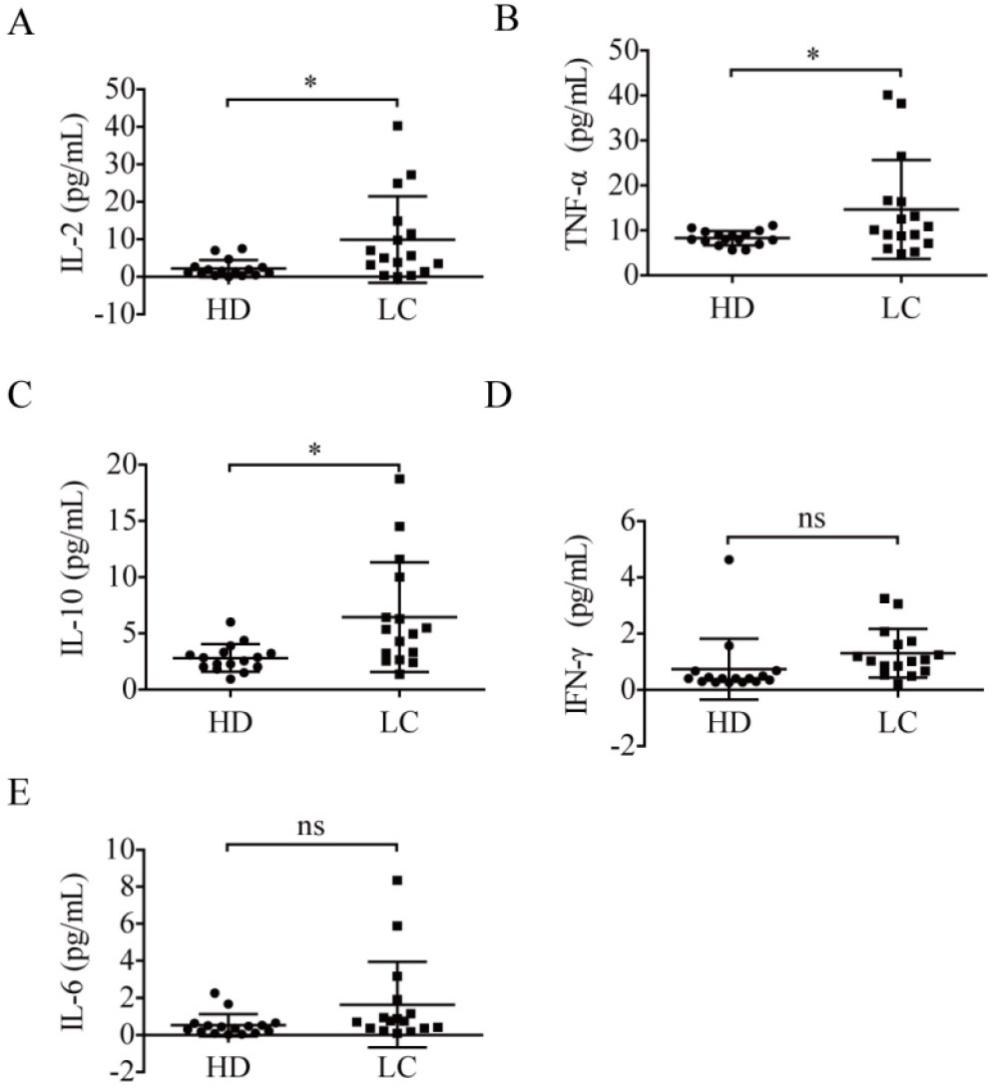
** Plasma cytokines in lung cancer patients.** Comparison of the concentrations of IL-2 (**A**), TNF-α (**B**), IL-10 (**C**), IFN-γ (**D**), and IL-6 (**E**) in the plasma of 16 lung cancer patients (LC) and 16 healthy donors (HD). Data were analyzed and compared using the paired *t*-test; *p < 0.05; ns: not significant.

To analyze the relationship between PD-1^+^ NK cells and plasma cytokine concentrations, the plasma cytokine expression levels of all participants were detected. As a result, compared to the healthy donors, the patients exhibited higher levels of plasma IL-2 (9.92 ± 11.55 pg/mL *vs.* 2.21 ± 2.28 pg/mL, *p* = 0.0137; Fig. 4A), TNF-α (14.67 ± 10.97 pg/mL *vs.* 8.30 ± 1.60 pg/mL, *p* = 0.0288; Fig. 4B), and IL-10 (6.45 ± 4.87 pg/mL *vs.* 2.82 ± 1.22 pg/mL, *p* = 0.0070; Fig. 4C). However, the expression levels of IFN-γ (1.31 ± 0.87 pg/mL *vs.* 0.74 ± 1.09 pg/mL, *p* = 0.1135; Fig. 4D) and IL-6 (1.63 ± 2.31 pg/mL *vs.* 0.53 ± 0.60 pg/mL, *p* = 0.0750; Fig. 4E) in the patients were not significantly different from those of healthy donors.

**Figure 5:**
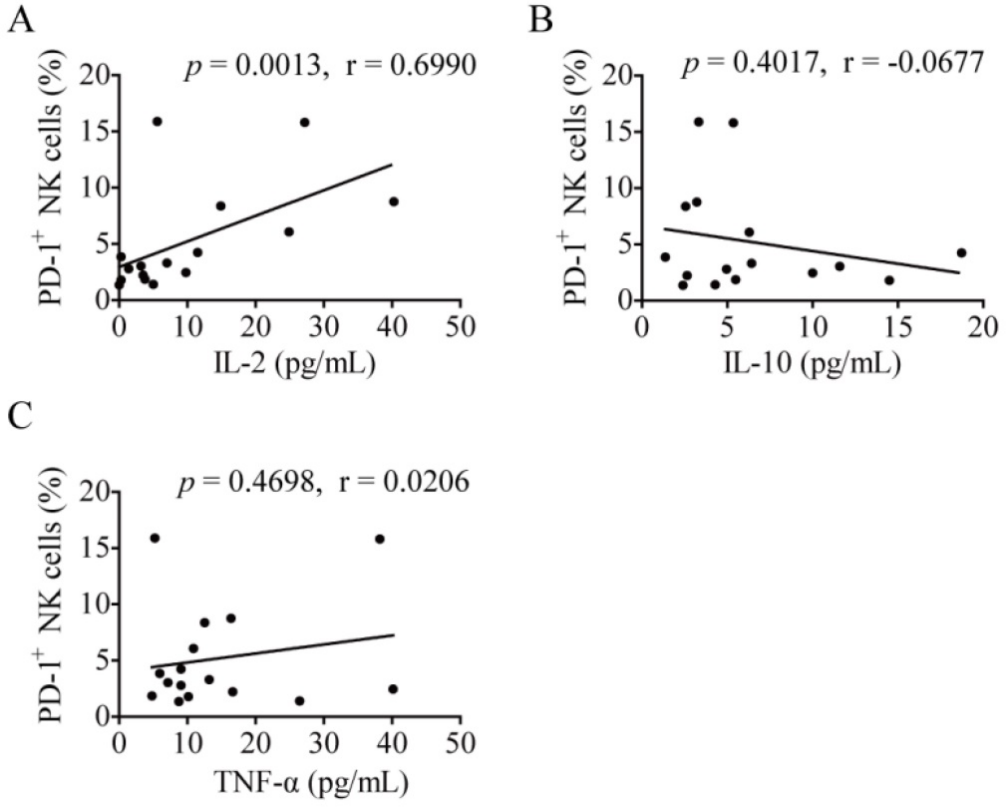
** The correlation between plasma cytokine concentration and PD-1^+^ NK cells.** (**A-C**) The plasma concentrations of IL-2, IL-10, and TNF-α of lung cancer patients (LC) were determined by the Miliplex human cytokine/chemokine 96-well plate assay. Correlation analysis between plasma cytokine concentration and PD-1^+^ NK cells in LC was performed using Spearman's test.

As for the correlation between plasma cytokine concentration and PD-1^+^ NK cells in lung cancer, a correlation analysis was conducted. The level of PD-1^+^ NK cells was significantly positively related to the concentration of plasma IL-2 (*p* = 0.0013, r = 0.6990; Fig. 5A). However, there was no relation between the percentages of PD-1^+^ NK cells and the concentration of plasma IL-10 (*p* = 0.4017, r = -0.0677; Fig. 5B) or TNF-α (*p* = 0.4698, r = 0.0206; Fig. 5C).

### IL-2 increased NK cell PD-1 expression

**Figure 6:**
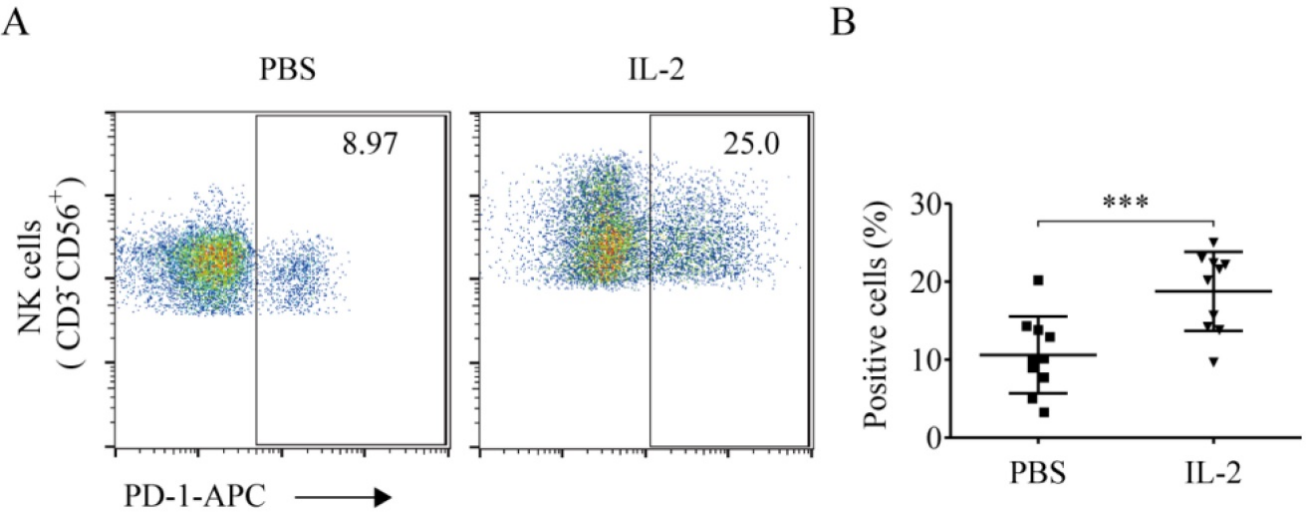
** IL-2 enhanced PD-1 expression on NK cells.** After treatment with PBS and 200 IU/mL IL-2 for 48 h, peripheral blood mononuclear cells (PBMCs) were harvested and stained with CD56, CD3, and PD-1. (**A**) Representative flow cytometry analysis of the expression of PD-1 on NK cells cultured with PBS and IL-2 from one representative lung cancer patient. (**B**) Comparison of the expression of PD-1 on NK cells cultured with PBS and IL-2 from ten lung cancer patients. Data were analyzed and compared using the paired *t*-test; ***p < 0.001; ns: not significant.

Compared to the PBS treatment group, IL-2 significantly elevated the ratio of PD-1^+^ NK cells (18.89% ± 5.21% *vs.* 10.62% ± 4.93%, *p* < 0.001). This demonstrated that IL-2 could enhance the ratio of PD-1^+^ NK cells (Fig. 6A and 6B).

## Discussion

NK cells are natural immune cells, which perform an important function in tumor immunosurveillance 28, 29. Solid tumors are reportedly characterized by decreased cell numbers and functional defects in NK cells 17, 30. We found that the antitumor function of NK cells in the peripheral blood of lung cancer patients was diminished compared to that in the healthy control donors, and the PD-1^+^ NK cell ratio in the lung cancer patients greatly exceeded that of the healthy individuals. More importantly, PD-1^+^ NK cells were less potent in secreting IFN-γ and had lower antitumor effects than PD-1^-^ NK cells, suggesting that the accumulation of PD-1^+^ NK cells in lung cancer may be responsible for the dysfunction of NK cells. Although, the features of PD-1 in T cells have been thoroughly analyzed 31-34, its features on NK cells have not been explored extensively, and some findings from different tumor types are controversial. Some studies have shown that PD-1^+^ NK cells are functionally defective 19, 20, 35, whereas results of others suggest that they are activated NK cells 36, 37. In lung cancer, we found that perforin, granzyme B, IFN-γ, and CD107a were lower in PD-1^+^ NK cells than those in PD-1^-^ NK cells. Hence, our findings support the theory that high PD-1 expression on NK cells counteracts the antitumor function of NK cells.

CD56^dim^ and CD56^bright^ NK cells are two subsets of NK cells. We observed that PD-1 was mainly expressed on the surface of CD56^dim^ NK cells from the patients of lung cancer, which is consistent with the findings in ovarian cancer and non-tumor infected patients 38. In esophageal squamous cell carcinoma, the increased proportion of PD-1^+^ NK cells has been related to poor prognosis 20. Therefore, we wanted to explore whether the enhanced percentage of peripheral blood PD-1^+^ NK cells could be used as a prognostic indicator for lung cancer. Unfortunately, due to inadequate numbers of participants involved and insufficient observation time in this study, the relationship between PD-1^+^ NK cells and lung cancer prognosis could not be determined. Therefore, further studies should widen the scope of this meaningful comparative analysis.

The expression of PD-1 on the T cell surface can be mediated by cytokines, such as IL-2 and IL-10 39, 40. Next, we asked whether the enhancement of PD-1^+^ NK cells in lung cancer associated with plasma cytokine. We demonstrated that the levels of IL-2, TNF-α, and IL-10 in the plasma from lung cancer patients were significantly higher than those in the plasma from healthy donors. Notably, in lung cancer, the concentration of plasma IL-2 was positively related with the proportion of PD-1^+^ NK cells in the blood. Moreover, *in vitro* studies have further indicated that IL-2 can quickly and significantly increase the PD-1^+^ NK cell ratios of lung cancer patients; and similar results been confirmed in NK cells from healthy donors 22. This suggests that the increased concentration of IL-2 in plasma from lung cancer patients may be one of the underlying reasons for the generation of PD-1^+^ NK cells in blood.

Given that the expression of CD107a has been considered as a sensitive marker for determination of the cytotoxic activity 41, to assess the cytotoxic activity of PD-1^+^ NK cells, we performed CD107a tests, rather than a cytotoxicity assay of NK cells on tumor cells. Insufficient quantity of PD-1^+^ NK cells from patients had been the primary limitation of the present study. Although the proportion of PD-1^+^ NK cells in lung cancer patients is higher than that in healthy individuals, it is still relatively low. To get enough PD-1^+^ NK cells for detection of cytotoxicity, approximately 1 L of peripheral blood per patient is needed, which is beyond the scope of responsible patient care. In future research, we plan to construct a NK cell line expressing PD-1 and conduct a thorough research on the antitumor effect of PD-1^+^ NK cells and the mechanisms underlying the PD-1 regulation of NK cell function.

## Conclusion

In conclusion, this study reveals a subset of NK cells with high expression of PD-1 in the blood of the patients with lung cancer, and the abundant of these PD-1^+^ NK cells may be related to the increased concentration of IL-2 in the plasma of the patients. Moreover, the antitumor activity of PD-1^+^ NK cells is lower than that of PD-1^-^ NK cells. These results further support the notion that PD-1 downregulates the antitumor effect of not only T cells but also NK cells. Given the importance of NK cells in antitumor immunity, our work provides a rationale for blocking the PD-1 and PD-L1 axis to rescue the dysfunction of NK cells in cancers including lung cancer.

## Figures and Tables

**Table 1:** Characteristics of lung cancer patients

Patient No.	Sex	Age (years)	Type of Tumor	Stage of Disease
1	Male	70	Adenocarcinoma	T2aN2M0 IIIA
2	Female	66	Adenocarcinoma	T4N2M1a IV
3	Male	52	Adenocarcinoma	T2aN2M0 IIIA
4	Male	40	Squamous carcinoma	T2aN2M0 IIIA
5	Male	63	Adenocarcinoma	T2N2M1 IV
6	Male	60	Adenocarcinoma	T3N3M0 IIIB
7	Female	51	Adenocarcinoma	T4N2M0 IIIB
8	Male	68	Adenocarcinoma	T4N2M1a IV
9	Female	65	Adenocarcinoma	T2aN2M0 IIIA
10	Female	45	Adenocarcinoma	T2aN2M0 IIIA
11	Female	62	Adenocarcinoma	T2aN2M1b IV
12	Male	43	Adenocarcinoma	T4N2M0 IIIB
13	Female	53	Adenocarcinoma	T2aN2M0 IIIA
14	Male	53	Adenocarcinoma	cT2aN2M0 IIIA
15	Male	48	Adenocarcinoma	pT2N0M0 IB
16	Male	52	Adenocarcinoma	pT2bN1M0 IIB

**Table 2:** Characteristics of healthy donors

Donor No.	Sex	Age (years)
1	Female	65
2	Female	67
3	Male	48
4	Male	52
5	Male	59
6	Female	45
7	Female	70
8	Male	69
9	Male	50
10	Male	61
11	Female	50
12	Female	57
13	Female	48
14	Male	57
15	Male	46
16	Male	54

## Abbreviations

NK: natural killer
IL: interleukin
PD-L1: programmed cell death ligand-1
PD-1: programmed cell death 1
